# Supplementary material for: Long-term effectiveness and tolerability of galcanezumab in patients with migraine excluded from clinical trials: real world evidence of 1055 patients with 1 year follow-up from the Galca-Only registry
Source: J Headache Pain. 2023 Nov 22;24(1):157. doi: 10.1186/s10194-023-01690-2 (PMC10666410; doi:10.1186/s10194-023-01690-2)
Supplement: Supplementary file 1 — Additional file1: Supplementary Table 1. Comparison in headache days per month during all the timepoints between patients with and without each subgroup. Supplementary Table 2. Logistic regression analysis of the variables that were associated with a 50% responder rate between weeks 20-24. Values in bold denote statistical significance. Supplementary Table 3. Linear regression analysis about the variables associated with a different change in headache days per month between weeks 20-24, compared with the baseline. Analysis done per intention-to-treat. Supplementary Table 4. Linear regression analysis about the variables associated with a change in headache days per month between weeks 44-48 compared with the baseline. Analysis done per intention-to-treat. [file 10194_2023_1690_MOESM1_ESM.docx]

**Long-term effectiveness and tolerability of galcanezumab in patients with migraine excluded from clinical trials: Real Wold Evidence of 1055 patients with 1 year follow-up from the Galca-Only registry.**

**Authors:**

Victor Obach, MD^1^, Fernando Velasco, MD, PhD^2^, Rocio Alvarez Escudero, MD^3^,María Martín Bujanda, MD^4^, Sonsoles Aranceta, MD^5^, Neus Fabregat, MD^1^, Teresa Marco, SN^1^, Aintzine Ruisanchez, MD^6^, Natalia Roncero, MD^7^, Ane Mínguez, MD^8^, Marta Ruibal, MD^8^, Daniel Guisado-Alonso, MD^9^; Antia Moreira, MD, PhD^9^, Elisa Cuadrado-Godia, MD, PhD^9^.Amaya Echeverria, MD^10^, Izaro Kortazar Zubizarreta, MD^10^, Alba López-Bravo, MD^11^, Nuria Riesco, MD, PhD^3^, Lucia González-Fernández, MD^3^, Nuria Pola, MD^1^, Paula Manera, MS^12^, Ángel Luis Guerrero-Peral, MD, PhD^13^, Agustín Oterino Duran, MD, PhD^3^, Yésica González-Osorio, PM^13^, Rosario Armand, MD^1^, Santiago Fernández-Fernández, MD, PhD^1^, David García-Azorín, MD, PhD^13^, Juan Carlos García-Moncó, MD, PhD^7^

1- Headache Unit. Neurology Department. Hospital Clinic. Univesitat de Barcelona. IDIBAPS. Barcelona. Spain.

2- Neurology Department. Hospital Universitario Cruces. Bilbao. Spain.

3- Neurology Department. Hospital Universitario Central de Asturias. Oviedo, Spain.

4- Neurology Department. Hospital Universitario de Navarra. Spain.

5- Neurology Department. Hospital Parc Tauli. Sabadell. Barcelona. Spain

6- Neurology Department. Hospital Universitario de Galdakao-Usansolo. Bilbao. Spain.

7- Neurology Department. Hospital Universitario de Basurto. Bilbao. Spain

8- Neurology Department. Hospital Universitario de Donostia. San Sebantian. Spain

9- Neurology Department. Hospital del Mar. Department of Medicine and Life

Sciences, Universitat Pompeu Fabra (UPF), Barcelona, Spain.

10- Neurology Department. Araba University Hospital. Bioaraba. Osakidetza Basque Health Service. Spain.

11- Headache Unit, Neurology Department. Hospital Reina Sofía, Tudela de Navarra. Aragon Institute for Health Research (IIS Aragón), Zaragoza. Spain.

12- Faculty of Health and Life Sciences. Universitat Pompeu Fabra. Barcelona. Spain.

13- Headache Unit. Neurology Department. Hospital Clinico Universitario. Department of Medicine, University of Valladolid. Spain

**Correspondence to:**

David García-Azorín. Address: Headache Unit, Hospital Clínico Universitario de Valladolid. Calle Rondilla Sta.Teresa, s/n, 47010 Valladolid. Tel: +34665872228. E-mail address: [dgazorin@ucm.es](mailto:dgazorin@ucm.es)

**Abstract**

**Background**: Galcanezumab has shown efficacy and effectiveness in the treatment of episodic and chronic migraine (CM), however, the population represented in randomized clinical trials (RCTs) differs from the population observed in real-world setting. To describe the long-term effectiveness and tolerability of galcanezumab in clinical practice in patients excluded from RCTs.

**Methods:** Multicenter prospective cohort study of consecutive patients with chronic and high-frequency episodic migraine (HFEM) with prior failure to three or more migraine preventive drugs, treated with galcanezumab and followed up for 12 months.

**Results**: We enrolled 1055 patients, aged 50 (IQR: 42-58), 82.9% female, 76.4% chronic migraine, 69% with at least one exclusion criteria for RCTs, including age >65 (n=121), concomitant use of onabotulinumtoxinA (n=185), daily headache at baseline (n=347), chronic painful syndromes (n=206), fibromyalgia (n=101) or treatment resistance (n=957). The median number of prior preventive treatments was 4 (IQR: 3-5). The retention rate was 90.8%, 76.8% and 71.4% at 3, 6 and 12 months. The main reasons for treatment discontinuation were lack of effectiveness (21.1%) and inadequate tolerability (6.6%).

The 30%, 50% and 75% responder rates were 62.6%, 49.8% and 24.2% between weeks 8-12; 60.9%, 48.8% and 24.6% between weeks 20-24; and 59.7%, 48.3% and 24.6% between weeks 44-48. Daily headache at baseline (OR: 0.619; 95%CI: 0.469-0.817) and patient’s age (OR: 1.016; 95%CI: 1.005-1.026) were associated with 50% response at weeks 20-24. The variables that were associated with a higher reduction of headache days between weeks 20-24 were patient’s age (0.068; 95% CI: 0.018-0.119) and headache days per month at baseline (0.451; 95% CI: 0.319-0.583), while psychiatric comorbidity (-1.587; 95% CI: -2.626—0.538) and daily headache at baseline (-2.718; 95% CI: -4.58—0.869) were associated with fewer reduction in the number of headache days between weeks 20-24.

**Conclusion:**

This study provides class III evidence of effectiveness and tolerability of galcanezumab in patients with HFEM and CM with comorbidities that would result in exclusion of the pivotal RCTs. Nonetheless, the clinical results over a 12-month period were similar to the efficacy observed in randomized controlled trials. Few patients discontinued the drug due to inadequate tolerability.

**Keywords**: Migraine, Monoclonal antibody, CGRP, Galcanezumab, Elderly, Fibromyagia, Daily headache

**Introduction**

Galcanezumab is a monoclonal antibody (mAb) targeting the calcitonin gene-related peptide (CGRP) that has shown efficacy and tolerability in the treatment of episodic and chronic migraine (CM) [1-7]. Evidence from randomized controlled trials (RCTs) is limited to 3 [3,5,6] or 6-month follow-up [1-2], including n=425 [1], n=454 [2], n=529 [4], and n=232 [6] galcanezumab-treated patients, respectively. Twelve-month open-label studies have mainly focused on tolerability [4,5]. Real-world evidence is based on short-term follow-up and small sample sizes, coming from Korea (n=87, 3-month follow-up) [8], Japan (n=52, 3-month follow-up) [9], and Italy (n=163, n=771, 6-month follow-up; and n=191, 12-month follow-up) [10-12].

The pivotal trials excluded patients older than 65 years, patients with migraine onset after 50 years, persistent daily headache, head or neck trauma, other primary headache disorders, concomitant or recent (<30 days) preventive treatment, prior failure to >3 medication classes, serious or unstable medical or psychiatric conditions, or patients at risk for acute cardiovascular events based on history or ECG [1-7].

We aimed to provide real-world evidence about long-term effectiveness and tolerability of galcanezumab in a large sample of patients, many of whom would have been excluded from RCTs, including age over 65 years old, daily headache at baseline, concomitant preventive treatment, treatment resistance, other painful syndromes and/or fibromyalgia

**Methods**

*Study setting*

Galca-only consortium comprises twelve Spanish public university headache centers where galcanezumab was the only available CGRP-mAb. The study period was from November 15, 2019, to time of last included patient on January 31, 2022, and follow up data was closed on April 15, 2023.

*Study design*

A prospective cohort study was conducted, following the Guidelines of the International Headache Society for Clinic-Based Headache Registries [13], and was reported according to the Strengthening of the Reporting in Observational Studies in Epidemiology (STROBE) statement [14]. The study was approved by the Hospital Clinic of Barcelona Ethics Committee (HCB/2021/1327).

*Participants*

The inclusion criteria were: 1) diagnosis of migraine, according to the International Classification of Headache Disorders, 3rd version [15]; 2) high-frequency episodic migraine- HFEM (at least eight migraine days per month) or CM in the preceding three months; 3) treatment with galcanezumab according to pre-specified reimbursement criteria for mAb of the Spanish Health authorities, which included prior failure to 3 oral preventive treatments being one of these OnabotA in the case of CM patients, defined as insufficient effectiveness and/or inadequate tolerability.

Patients were excluded if they 1) were younger than 18 years old; or 2) did not sign the informed consent form.

All consecutive migraine patients were screened for eligibility. Patients were quarterly assessed by in-person clinical evaluations conducted by a headache expert and completed headache diaries from the three months preceding the treatment until the twelfth month of treatment.

*Drug administration*

A 240 mg-loading dose of galcanezumab was administered, followed by a 120 mg monthly dose. The concomitant use of drugs with preventive effect for migraine was allowed if resulted ineffective and were stable in the preceding three months.

*Study outcomes*

The study outcomes were selected based on the Guidelines of the International Headache Society for controlled trials of preventive treatment of chronic migraine in adults [16]. The primary study outcome was the change in the number of headache days per month (HDM), assessed at weeks 20-24, compared to the average HDM of the three months prior to the treatment onset. Secondary outcomes included the change in the number of HDM between weeks 8-12 and 44-48. The 30%, 50% and 75% responder rates were estimated between weeks 8-12, 20-24, and 44-48. Responder rates were calculated as percent reduction from baseline in the number of HDM in each treatment period, and were calculated both per intention-to-treat (ITT) and per protocol (PP). The retention rate of galcanezumab and the reasons for discontinuation were assessed.

*Data source and measurements*

Data was prospectively collected from headache diaries. In the initial kick-off meeting of the study with the participating researchers, the definition of all the variables selected in the registry were discussed and adopted by all participants. Regular follow-up meetings were conducted every 2-3 months to ensure the study protocol adherence. Any doubts from any center during the study were centralized by two of the authors (VO and DGA) and shared with all the consortium members. The data collection elements were harmonized. A series of demographic and clinical variables were gathered, including age at treatment onset, sex, prior history of psychiatric disorders, other chronic pain conditions including fibromyalgia, type of migraine (HFEM or CM), years of migraine, prior number of preventive treatments, treatment resistant migraine criteria [7]~~,~~ and concomitant use of migraine preventive drugs. Frequency and type of adverse effects leading to treatment discontinuation was assessed. Patients collected the information by using headache diaries, with consistent definitions within the different participating sites.

*Bias, confounders, and effect modifiers*

Selection bias was avoided by screening a cohort of consecutive, unselected patients. The outcomes of interest were not present at the study onset. Follow-up was based on headache diaries, avoiding any subjective or biased evaluation. A 12-month follow-up was completed for all enrolled subjects, and treatment discontinuation or loss of patients was an unlikely source of bias due to the conservative approaches in the outcome evaluation and analysis. The effect of confounders was addressed in the statistical analysis.

*Study size*

There was no formal sample size calculation. Considering that the largest the pivotal RCT included 555 [5] patients, a larger sample size was deemed optimal.

*Data analysis*

Qualitative variables are presented as frequency and percentage, and quantitative variables as mean and standard deviation (SD) or median and inter-quartile range (IQR). Normality of the sample was assessed with the Kolmogorov-Smirnov test. Paired samples Student t-test and Wilcoxon test were used. To evaluate which variables were associated with a 50% responder rate between weeks 20-24, a univariate logistic regression was done, selecting as dependent variable the presence of a 50% response between weeks 20-24. In addition, to assess which variables were associated with a higher/fewer reduction of headache days per month between weeks 20-24 and 44-48, two linear regression analyses were done, including as dependen variable the change in headache days per month. All variables with a P value <0.2 were included in a multivariate regression analysis. Odds ratios (OR) are presented together with the 95% confidence intervals (CI). Missing data and loss to follow-up were handled by conservative assumptions. In the case of evolutionary variables, both baseline carried-forward (BCF) and last-observation carried-forward (LOCF) were used [17]. The statistical significance threshold was set at 0.05, and multiple comparisons were managed by False Discovery Rate adjustment according to the Benjamini-Hochberg method [18]. The statistical analysis was done with SPSS v25.0 (IBM Corp, Armonk).

**Results**

A total of 1055 patients were included, aged 50 (IQR: 42-58) years, and 876 (82.9%) women. Regarding comorbidities, 441 (41.8%) patients had at least one comorbidity, including prior history of psychiatric diseases 346 (32.8%), other painful syndromes 206 (19.5%), or fibromyalgia 101 (9.6%). Migraine type corresponded to CM in 806 (76.4%) and HFEM in 249 (23.6%). The mean duration of HFEM or CM was 7 (IQR: 4-12) and 8 (IQR: 5-15) years. The median number of prior preventive treatments was 4 (IQR: 3-5), 957 (90.7%) patients fulfilled criteria of treatment resistant migraine and 328 (31.1%) had failed to five or more preventive treatments. At baseline, 185 (17.5%) patients were under onabotulinumtoxinA. A total of 729 (69.1%) patients had at least one contraindication listed in the pivotal RCTs. Table 1 shows the differences within the different subgroups.

Table 1: Baseline data in the entire study sample and the study subgroups

|  | Entire study sample (n=1055) | Age >65 (n=121) | OnabotA use (n=185) | Daily headache (n=347) | Chronic pain (n=206) | Fibromyalgia (n=101) | Treatment resistant (n=957) |
| --- | --- | --- | --- | --- | --- | --- | --- |
| Age (years) | 50 [42-58] | 68 [66-72] | 50 [43-57] | 51 [43-59] | 51 [45-59] | 50 [43-58] | 50 [42-58] |
| Female sex (n, %) | 875 (82.9%) | 89 (81%) | 164 (88.6%) | 289 (85.9%) | 183 (88.8%) | 97 (96%) | 793 (82.9%) |
| Chronic migraine (n, %) | 806 (76.4%) | 94 (77.7%) | 144 (77.8%) | 333 (96%) | 166 (80.6%) | 86 (85.1%) | 740 (77.3%) |
| Duration of migraine (years) | 8 [4-14] | 11 [6-20] | 6 [4-10] | 7 [4-13] | 8 [5-15] | 8 [4-15] | 8 [4-14] |
| Prior number of preventives | 4 [3-5] | 4 [3-5] | 4 [3-5] | 4 [3-5] | 4 [3-5] | 4 [3-5.5] | 4 [3-5] |
| Psychiatric disorders (n, %) | 346 (32.8%) | 35 (28.9%) | 75 (40.5%) | 130 (37.5%) | 124 (60.2%) | 70 (69.3%) | 308 (32.2%) |
| Chronic pain (n, %) | 206 (19.5%) | 26 (21.5%) | 39 (21.1%) | 91 (26.2%) | 206 (100%) | 69 (68.3%) | 182 (19%) |
| Fibromyalgia (n, %) | 101 (9.6%) | 10 (8.3%) | 28 (15.1%) | 47 (13.5%) | 69 (33.5%) | 101 (100%) | 87 (9.1%) |
| Headache days per month at baseline | 20 [14-30] | 25 [15-30] | 20 [15-30] | 30 [30-30] | 27 [17-30] | 27 [20-30] | 20 [14-30] |

*Persistence, tolerability, and reasons for discontinuation*

The retention rate was 958 (90.8%) at month-3, 810 (76.8%) at month-6 and 753 (71.4%) at month-12. Treatment discontinuation was attributed to lack of effectiveness in 60 (5.7%) patients at month-3, in 181 (17.1%) patients at month-6, and in 223 (21.1%) at month-12. Discontinuation was attributed to an inadequate tolerability in 37 (3.5%) patients at month-3, in 55 (5.2%) patients at month-6, and in 70 (6.6%) patients at month-12. Table 2 shows the frequency and type of adverse events leading to treatment discontinuation. Nine (0.9%) patients discontinued galcanezumab due to other reasons, such as pregnancy desire or change of city.

Table 2: Adverse events leading to treatment discontinuation

Seventy patients (6.6%) discontinued galcanezumab due to 105 different adverse events.

| **Adverse event** | **N (%)** |
| --- | --- |
| Dizziness | 19 (1.8) |
| Constipation | 13 (1.2) |
| Vertigo | 12 (1.1) |
| Localized cutaneous rash | 9 (0.8) |
| Injection site pain | 9 (0.8) |
| Generalized cutaneous rash | 7 (0.6) |
| Asthenia | 5 (0.5) |
| Arterial hypotension | 3 (0.3) |
| Drowsiness | 3 (0.3) |
| Confusion | 3 (0.3) |
| Diarrhea | 3 (0.3) |
| Nausea | 3 (0.3) |
| Paresthesia | 3 (0.3) |
| Generalized pain | 3 (0.3) |
| Anxiety | 2 (0.2) |
| Increased weight | 2 (0,2) |
| Anorexia | 1 (0.1) |
| Tinnitus | 1 (0.1) |
| Blurred vission | 1 (0.1) |
| Abdominal pain | 1 (0.1) |
| Blood pressure instability | 1 (0.1) |
| Headache worsening | 1 (0.1) |

*Response to treatment*

There was a statistically significant reduction in the number of HDM, compared with baseline, at all timepoints, both in the PP and ITT analyses (table 3). Figure 1 shows the 30%, 50% and 75% responder rates at weeks 8-12, 20-24 and 44-48. Patients with EM had higher responder rates than patients with CM (table 4).

Table 3: Median reduction of headache days per month at different time intervals

|  | Weeks 8-12 | FDR-adjusted *P* value | Weeks 20-24 | FDR-adjusted *P* value | Weeks 44-48 | FDR-adjusted *P* value |
| --- | --- | --- | --- | --- | --- | --- |
| PP | 8 [2-13] | **<0.001** | 9 [3-15] | **<0.001** | 10 [5-16] | **<0.001** |
| ITT-BCF | 7 [1-13] | **<0.001** | 7 [0-13] | **<0.001** | 7 [0-14] | **<0.001** |
| ITT-LOCF | 7 [1-13] | **<0.001** | 8 [0-14] | **<0.001** | 7 [0-14] | **<0.001** |

PP: Per protocol. ITT: Per intention-to-treat. BCF: Baseline carried forward. LOCF: Last observation carried forward. Values in bold denotes statistical signification. FDR: False discovery rate.

Figure 1. Response rates at different time intervals. The two types of analyses conducted in patients who discontinued treatment are shown: BCF: baseline carried forward, LOCF: last observation carried forward.


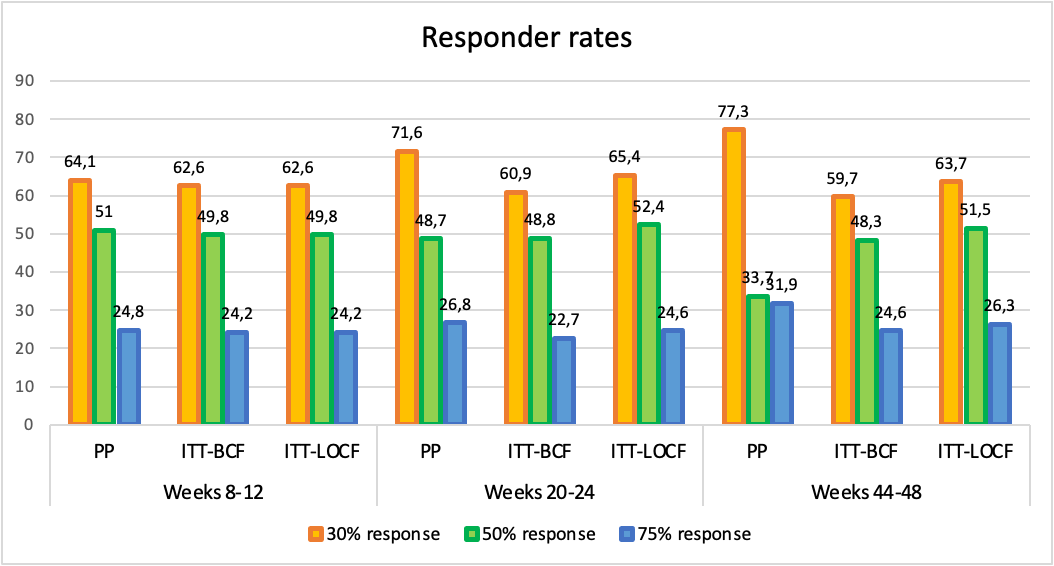


Table 4: Responder rates in the entire study sample, chronic migraine patients and high-frequency episodic migraine patients at different time intervals.

|  | Entire study sample (n=1055) (%) | Chronic migraine (n=806) (%) | Episodic migraine (n=249) (%) | FDR-adjusted *P* value |
| --- | --- | --- | --- | --- |
| *Weeks 8-12* | | | | |
| 30%R PP (n=958) | 64.1 | 59.3 | 79.8 | **<0.001** |
| 30%R ITT BCF (n=1055) | 62.6 | 57.9 | 77.5 | **<0.001** |
| 30%R ITT LOCF (n=1055) | 62.6 | 57.9 | 77.5 | **<0.001** |
| 50%R PP (n=958) | 51.0 | 47.1 | 63.6 | **<0.001** |
| 50%R ITT BCF (n=1055) | 49.8 | 46.0 | 61.8 | **<0.001** |
| 50%R ITT LOCF (n=1055) | 49.8 | 46.0 | 61.8 | **<0.001** |
| 75%R PP (n=958) | 24.8 | 22.3 | 32.6 | **0.001** |
| 75%R ITT BCF (n=1055) | 24.2 | 21.8 | 31.7 | **0.001** |
| 75%R ITT LOCF (n=1055) | 24.2 | 21.8 | 31.7 | **0.001** |
| *Weeks 20-24* | | | | |
| 30%R PP (n=810) | 71.6 | 68.8 | 80.4 | **0.001** |
| 30%R ITT BCF (n=1055) | 60.9 | 57.8 | 70.7 | **<0.001** |
| 30%R ITT LOCF (n=1055) | 65.4 | 61.0 | 79.5 | **<0.001** |
| 50%R PP (n=810) | 48.7 | 54.6 | 66.2 | **0.003** |
| 50%R ITT BCF (n=1055) | 48.8 | 45.9 | 58.2 | **0.001** |
| 50%R ITT LOCF (n=1055) | 52.4 | 48.4 | 65.5 | **<0.001** |
| 75%R PP (n=810) | 26.8 | 25.4 | 31.1 | 0.104 |
| 75%R ITT BCF (n=1055) | 22.7 | 21.3 | 27.3 | 0.056 |
| 75%R ITT LOCF (n=1055) | 24.6 | 22.5 | 31.7 | **0.003** |
| *Weeks 44-48* | | | | |
| 30%R PP (n=753) | 77.3 | 75.9 | 81.4 | 0.200 |
| 30%R ITT BCF (n=1055) | 59.7 | 56.2 | 71.1 | **<0.001** |
| 30%R ITT LOCF (n=1055) | 63.7 | 60.5 | 79.1 | **<0.001** |
| 50%R PP (n=753) | 33.7 | 60.5 | 69.3 | 0.079 |
| 50%R ITT BCF (n=1055) | 48.3 | 44.5 | 60.6 | **<0.001** |
| 50%R ITT LOCF (n=1055) | 51.5 | 46.7 | 67.1 | **<0.001** |
| 75%R PP (n=753) | 31.9 | 32.0 | 31.4 | 0.898 |
| 75%R ITT BCF (n=1055) | 24.6 | 23.0 | 30.1 | **0.033** |
| 75%R ITT LOCF (n=1055) | 26.3 | 23.8 | 34.1 | **0.001** |

PP: Per protocol. ITT: Per intention-to-treat. BCF: Baseline carried forward. LOCF: Last observation carried forward. Values in bold denotes statistical signification. FDR: False discovery rate.

*Response to treatment between the different subgroups*

Figure 2 shows the headache days per month at baseline and all time points according to the ITT analysis between the different subgroups. There were statistically significant differences in patients with concomitant OnabotA use between weeks 8-12 and 20-24; and with patients with daily headache, chronic pain, and fibromyalgia at all time points (supplementary table 1). Figure 3 shows the responder rates at all time points according to the ITT analysis.

Figure 2. Headache days per month at baseline, weeks 8-12, weeks 20-24 and weeks 44-48 in the comparison between patients A) aged 65, B) concomitant use of OnabotA, C) daily headache at baseline, D) chronic painful syndromes, E) Fibromyalgia, and F) treatment resistance. Missing data is presented as baseline carried forward.

Figure 3: Responder rate within the different groups according to the ITT analysis.


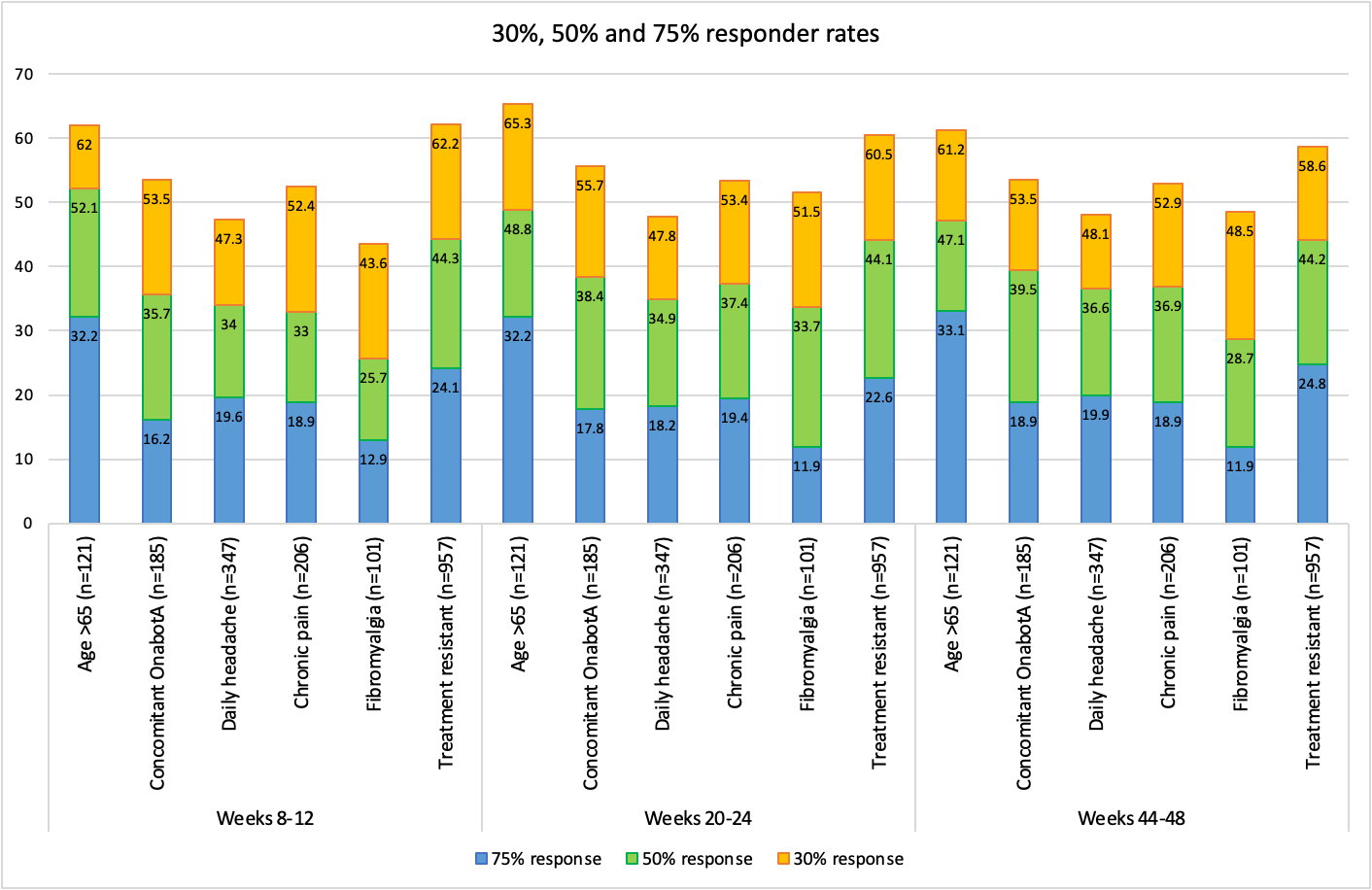


*Response predictors*

In the univariate logistic regression analysis (supplementary table 2), patients’ age, type of migraine, psychiatric comorbidity, and daily headache at baseline were associated with 50% responder rate between weeks 20-24. In the multivariate regression analysis, daily headache at baseline (OR: 0.619; 95%CI: 0.469-0.817) and patients’age (OR: 1.016; 95%CI: 1.005-1.026) remained statistically significant.

In the multivariate linear regression analysis (supplementary table 3), the variables that were associated with higher reduction of headache days between weeks 20-24 were patients’ age (regression coefficient: 0.068 (95% CI: 0.018 – 0.119); FDR-adjusted P=0.014) and headache days per month at baseline (regression coefficient: 0.451 (95% CI: 0.319 – 0.583); FDR-adjusted p<0.001), while psychiatric comorbidity (regression coefficient: -1.587 (95% CI: -2.636 - -0.538); FDR-adjusted p=0.009) and daily headache at baseline (regression coefficient: -2.718 (95% CI: -4.568 - -0.869); FDR-adjusted p=0.009) were associated with a fewer reduction of headache days per month.

When assessed between weeks 44-48 (supplementary table 4), headache days per month (regression coefficient: 0.473 (95% CI: 0.335 – 0.611), FDR-adjusted p<0.001) was associated with a higher reduction in headache days per month, while psychiatric comorbidity (regression coefficient: -1.932 (95% CI: -3.030 - -0.833); FDR-adjusted p=0.003), was associated with a fewer reduction of headache days per month.

**Discussion**

This study supports the effectiveness and tolerability of galcanezumab in migraine; it also provides hope for those people living with migraine who are ineligible for anti-CGRP mAbs based on the RCTs results. A correct diagnosis of migraine is key in adequately selecting patients who will benefit from this drug despite their comorbidities, and the reason for the high success in patients treated with anti-CGRP antibodies in real world conditions.

In this study, the first consecutive patients who began treatment with galcanezumab in twelve university public hospitals up to January 2022 are represented. This study is free from any form of selection bias, since galcanezumab was the only anti-CGRP mAb that was available at the participating sites. In addition, the treatment was subsidized by the national healthcare insurance in patients with HFEM or CM who previously failed to three or more preventive treatments. Patients were followed for a 12-month period, since this was the pre-specified treatment period in all study sites, given that not every site was allowed to extend the treatment for a longer duration without a previous “vacation” of treatment. The first remarkable finding of the study is that the population treated in a real-world setting significantly differs from the population represented in the RCTs. In this regard, 69% of patients would not be eligible for inclusion in RCTs. The proportion of screening-failure patients in the pivotal RCTs was 809/1671 (48.1%) in EVOLVE-1 study [1], 922/1696 (54.4%) in EVOLVE-2 [2], 786/1903 (41,3%) in REGAIN [4], and 121/610 (19.8%) in CONQUER [6].

The external validity of this study is high, and yet, we also aimed at a high internal validity. To this end, a conservative statistical analysis was performed, conducting analyses both per protocol and per intention-to-treat, and using as assumption the most conservative imputation methods, such as LOCF and BCF, assuming that if a measurement was not available, the baseline situation persisted [2]. To provide a better understanding of the clinical results, effectiveness was assessed at three different time points in all patients with various approaches [16,19].

Despite this, the clinical results did not differ significantly from RCTs. Table 5 shows the 50% and 75% responder rates observed in the RCTs and in the Galca-only study. It is notable that all responder rates from our study, according to the most conservative approach, were in line with those observed in the RCTs, except for the 75% responder rates, which were slightly lower than the results observed in the EVOLVE-1 [1] and EVOLVE-2 [1] studies. When compared with other real-world studies, our overall results were also similar (table 5) [20] .

Albeit the anti-CGRP mAbs were approved following the results of the initial pivotal RCTs, these are reimbursed only to treatment-resistant patients. It is currently a matter of debate whether these should be prescribed as first-line treatment. Recently, a study compared the effectiveness of erenumab between treatment naïve patients versus patients with the previous German reimbursement policy, that required the prior use and failure or contraindication to all prophylactic medication classes of first choice. In this group, the proportion of treatment naïve (which included a prior failure of 2 (IQR 1-2) treatments) patients who achieved a 50% responder rate was 63.5%, compared with 37.7% within patients with prior failure of 5 (IQR: 4-6) preventive treatments [24]. Our results suggest that galcanezumab may be also effective when used in treatment resistant patients and should be offered to these patients, however, future studies should explore whether the clinical benefit is higher in treatment naïve patients too.

Table 5: R50% and R75% responder rates from galcanezumab RCTs and real-world studies.

| **Study** | **3-months R50%** | **3-months R70%** | **6-months R50%** | **6-months R70%** |
| --- | --- | --- | --- | --- |
| EVOLVE-1 [1] (n=425) |  |  | 62.3% (120mg), 60.9% (240mg) | 38.8% (120mg), 38.5% (240mg) |
| EVOLVE-2 [2] (n=454) |  |  | 59.3% (120mg), 56.5% (240 mg) | 33.5% (120mg), 34.3% (240mg) |
| REGAIN [4] (n=529) | 27.6% (120mg), 27.5% (240mg) | 7.0% (120mg), 8.8% (240mg) |  |  |
| CONQUER [6] (n=232) | 41.8% (EM), 32.0% (CM) | 18.4% (EM), 8.8% (CM) |  |  |
| PERSIST [21] (n=260) | 54.9% | 29.2% |  |  |
| PERSIST [22]  open label (n=484) |  |  | 69.0% | 46.7% |
| Takizawa *et al* [9] (n=52) | 62% (all), 76% (EM), 48% (CM) | 35% (all), 40% EM, 30% (CM) |  |  |
| Vernieri *et al* [10] (n=245) | 79.4% (EM), 93.8% (CM) |  | 44.1% (EM), 40.6% (CM) |  |
| Kim *et al* [23] (n=104) | 55.7% |  |  |  |
| GALCA-ONLY (n=1055) | 49.8% (all), 61.8% (EM), 46% (CM) | 24.2% (all), 31.7% (EM), 21.8% (CM) | 48.8% (all), 58.2% (EM), 45.9% (CM) | 24.6% (all), 27.3% (EM), 21.3% (CM) |

The study by Kwon [8] et al was not represented because the study was conducted in the same site and the same study period as the study by Kim [23] et al. The study by Vernieri [11] et al is not represented since it reports MMD, which is not fully comparable to the reported data from this study.

Going to the specifics, in these subgroups, the two variables that were most clearly associated with a lower probability of response were daily headache at baseline and psychiatric comorbidity, as observed in another study that included 238 CM patients treated with galcanezumab for three months [25]. Although a higher frequency of headache days per month at baseline was associated with a better response to galcanezumab, this did not apply for patients with daily headache [25, 26]. This apparently paradoxical phenomenon may be explained by a different pathophysiology of daily migraine and requires further investigation. This could be related with a more “unreversible” state of the migraine disease or the absence of the cyclic nature of the disease, which could be partially explained by the changes in CGRP circulating levels during the attacks, being therefore less response to anti-CGRP therapies [7].

In contrast, other subgroups that traditionally have been associated with a lower probability of response could not be proven as such, when adjusted for multiple comparisons.

While the number of preventive drugs and chronic migraine has been associated with lower odds of response [8], these findings were not reproduced in our study except for daily headache when adjusted for multiple comparisons. Notably, our study had 10-times higher sample size and the comparisons were done by three different statistical models, which yield a more precise estimate. In another study [27], triptan response and BMI were associated with a higher probability of persistent response to galcanezumab; unfortunately, these parameters were not assessed in our study.

In the case of some specific subgroups, another study showed the effectiveness of various anti-CGRP mAbs in a series of 162 patients aged 65 or older, with a responder rates quite similar to the observed in our study [28]. In the case of the combination with OnabotA, two retrospective studies including 148 [29] and 257 [30] showed that the combination of OnabotA and anti-CGRP mAbs did not increase the risk of adverse events and showed an additional benefit in these cases, when compared to the pre-combination period. To this end, a RCT placebo-controlled, double-blind, double-dummy would be highly desirable to avoid any possible selection bias and other confounding factors.

Treatment persistence not only suggests an adequate tolerability, but a sufficient effectiveness. In our study only 6.6% patients discontinued galcanezumab due to poor tolerability. This numbers are in line with the observed in the RCTs, where 2-4% of patients discontinued the treatment due to an AE [1-6]. Thus, the combination of a notable effectiveness with a very good tolerability suggest that the optimal approach in patients pertaining to one of these subgroups, a treatment cycle with galcanezumab for at least three, ideally six months^12^ should be attempted. In a recent meta-analysis [31], patients treated with 120mg galcanezumab had 1.40 (95% CI: 1.16-1.70) higher odds of experiencing TEAE, but there was not an increased odds of adverse events leading to treatment discontinuation. The two AEs that had an increased odds in patients treated with galcanezumab 120 mg, compared with placebo were injection site erythema (OR: 1.87 (95% CI: 1.09-3.22) and injection site pruritus (OR: 13.48 (95% CI: 3.6-50.52) [28].

This study has some limitations. First, Headache Days per Month (HDM) were characterized and not Migraine Days per Month (MDM), to minimize the risk of misclassification of headache episodes. Second, as a real-world study, many patients presented multiple comorbidities and could be classified into various subgroups, which could influence the results and warrants a careful interpretation of these. Third, as in other pain and headache studies, the influence of placebo effect on these results cannot be excluded in observational study although the long-term 12-month follow-up results are less prone to this effect and the statistical analyses included conservative assumptions to avoid any falsely positive results.

**Conclusion**

This study provides class III evidence of the effectiveness and tolerability of galcanezumab in HFEM and CM patients in patients aged >65, concomitant onabotA, daily headache at baseline, other chronic painful syndromes, fibromyalgia and treatment resistance. A substantial proportion of patients excluded from the RCTs showed 30%, 50% and/or 75% responder rates, in the absence of serious TEAE. Future RCTs should consider not to exclude these populations, and galcanezumab should be considered as a potential treatment option by clinicians.

**References**

1. Stauffer VL, Dodick DW, Zhang Q, Carter JN, Ailani J, Conley RR (2018) Evaluation of Galcanezumab for the Prevention of Episodic Migraine: The EVOLVE-1 Randomized Clinical Trial. JAMA Neurol 75:1080-1088. doi: 10.1001/jamaneurol.2018.1212.
2. Skljarevski V, Matharu M, Millen BA, Ossipov MH, Kim BK, Yang JY (2018) Efﬁcacy and safety of galcanezumab for the prevention of episodic migraine: Results of the EVOLVE-2 Phase 3 randomized controlled clinical trial. Cephalalgia 38:1442-1454. doi: 10.1177/s0333102418779543.
3. Skljarevski V, Oakes TM, Zhang Q, Ferguson MB, Martinez J, Camporeale A, Johnson KW, Shan Q, Carter J, Schacht A, Goadsby PJ, Dodick DW (2018) Effect of Different Doses of Galcanezumab vs Placebo for Episodic Migraine Prevention: A Randomized Clinical Trial. JAMA Neurol 75:187-193. doi: 10.1001/jamaneurol.2017.3859.
4. Detke HC, Goadsby PJ, Wang S, Friedman DI, Selzler KJ, Aurora SK (2018) Galcanezumab in chronic migraine: The randomized, double-blind, placebo-controlled REGAIN study. Neurology 91:e2211-e2221. doi: 10.1212/WNL.0000000000006640.
5. Camporeale A, Kudrow D, Sides R, Wang S, Van Dycke A, Selzler KJ, Stauffer VL (2018) A phase 3, long-term, open-label safety study of Galcanezumab in patients with migraine. BMC Neurol 18:188. doi: 10.1186/s1288301811932.
6. Mulleners WM, Kim BK, Láinez MJA, Lanteri-Minet M, Pozo-Rosich P, Wang S, Tockhorn-Heidenreich A, Aurora SK, Nichols RM, Yunes-Medina L, Detke HC (2020) Safety and efﬁcacy of galcanezumab in patients for whom previous migraine preventive medication from two to four categories had failed (CONQUER): a multicentre, randomised, double-blind, placebo-controlled, phase 3b trial. Lancet Neurol 19:814-825. doi: 10.1016/s14744422(20)302799.
7. Sacco S, Braschinsky M, Ducros A, Lampl C, Little P, van den Brink AM, Pozo-Rosich P, Reuter U, de la Torre ER, Sanchez Del Rio M, Sinclair AJ, Katsarava Z, Martelletti P (2020) European headache federation consensus on the definition of resistant and refractory migraine : Developed with the endorsement of the European Migraine & Headache Alliance (EMHA). J Headache Pain 21:76. doi: 10.1186/s10194020011305.
8. Kwon S, Gil YE, Lee MJ (2022) Real-world efficacy of galcanezumab for the treatment of migraine in Korean patients. Cephalalgia 42:705-714. doi: 10.1177/s03331024221076481.
9. Takizawa T, Ohtani S, Watanabe N, Miyazaki N, Ishizuchi K, Sekiguchi K, Iba C, Shibata M, Takemura R, Hori S, Nakahara J (2022) Real-world evidence ofgalcanezumab for migraine treatment in Japan: a retrospective analysis. BMC Neurol 22:512. doi: 10.1186/s12883-022-03041-1.
10. Vernieri F, Altamura C, Brunelli N, Costa CM, Aurilia C, Egeo G, Fofi L, Favoni V, Pierangeli G, Lovati C, Aguggia M, d'Onofrio F, Doretti A, Di Fiore P, Finocchi C, Rao R, Bono F, Ranieri A, Albanese M, Cevoli S, Barbanti P; GARLIT Study Group (2021) Galcanezumab for the prevention of high frequency episodic and chronic migraine in real life in Italy: a multicenter prospective cohort study (the GARLIT study). J Headache Pain 22:35. doi: 10.1186/s10194021012471.
11. Vernieri F, Brunelli N, Marcosano M, Aurilia C, Egeo G, Lovati C, Favoni V, Perrotta A, Maestrini I, Rao R, d'Onofrio L, Finocchi C, Aguggia M, Bono F, Ranieri A, Albanese M, Di Piero V, Cevoli S, Altamura C, Barbanti P; GARLIT Study Group (2023) Maintenance of response and predictive factors of 1-year GalcanezumAb treatment in real-life migraine patients in Italy: The multicenter prospective cohort GARLIT study. Eur J Neurol 30:224-234.
12. Piero Barbanti, Cinzia Aurilia, Gabriella Egeo, Paola Torelli, Stefania Proietti, Sabina Cevoli, Stefano Bonassi, for the Italian Migraine Registry study group (2023) Neurology 101:482-488. DOI: 10.1212/WNL.0000000000207292
13. Schwedt TJ, Tassorelli C, Silberstein SD, Szperka CL, Kurth T, Pozo-Rosich P, Amin FM, Lipton RB, Dodick DW, Ashina M, Diener HC, Terwindt GM (2022) Guidelines of the International Headache Society for Clinic-Based Headache Registries, 1st dition. Cephalalgia 42:1099-1115. doi: 10.1177/s03331024221099035.
14. von Elm E, Altman DG, Egger M, Pocock SJ, Gøtzsche PC, Vandenbroucke JP; STROBE Initiative (2007) The Strengthening the Reporting of Observational Studies in Epidemiology (STROBE) statement: guidelines for reporting observational studies. Lancet 370:1453-7. doi: 10.1016/s01406736(07)61602-X.
15. Headache Classiﬁcation Committee of the International Headache Society (IHS) (2018) The International Classiﬁcation of Headache Disorders, 3rd edition. Cephalalgia 38:1-211. doi: 10.1177/s0333102417738202.
16. Diener HC, Tassorelli C, Dodick DW, Silberstein SD, Lipton RB, Ashina M, Becker WJ, Ferrari MD, Goadsby PJ, Pozo-Rosich P, Wang SJ, Houle TT, Hoek TCVD, Martinelli D, Terwindt GM. International Headache Society Clinical Trials Committee (2020) Guidelines of the International Headache Societyfor controlled trials of preventive treatment of migraine attacks in episodic migraine in adults. Cephalalgia 40:1026-1044. doi: 10.1177/s0333102420941839.
17. Shao J, Jordan DC, Pritchett YL (2009) Baseline observation carry forward: reasoning, properties, and practical issues. J Biopharm Stat 19:672-84. doi: 10.1080/s10543400902964118.
18. Benjamini Y, Hochberg Y (1995) Controlling the false discovery rate: A practical and powerful approach to multiple testing. J R Stat Soc Ser B 57:289–300.
19. Twisk JW, Rijnhart JJ, Hoekstra T, Schuster NA, Ter Wee MM, Heymans MW (2020) Intention-to-treat analysis when only a baseline value is available. Contemp Clin Trials Commun 20:100684. doi: 10.1016/j.conctc.2020.100684.
20. Ashina, S., Melo-Carrillo, A., Toluwanimi, A.et al (2023) Galcanezumab effects on incidence of headache after occurrence of triggers, premonitory symptoms, and aura in responders, non-responders, super-responders, and super non-responders. J Headache Pain 24: 26. doi.org/10.1186/s10194-023-01560-x
21. Hu B, Li G, Li X, Wu S, Yu T, Li X, Zhao H, Jia Z, Zhuang J, Yu S (2022) Galcanezumab in episodic migraine: the phase 3, randomized, double-blind, placebo-controlled PERSIST study. J Headache Pain 23:90. doi: 10.1186/s10194-022-01458-0.
22. Zhou, J., Zhong, L., Chowdhury, D et al (2023) Galcanezumab in patients with episodic migraine: results from the open-label period of the phase 3 PERSIST study. J Headache Pain 2:103. doi.org/10.1186/s10194-023-01613-1
23. Kim SA, Jang H, Lee MJ (2023) Predictors of galcanezumab response in a real-world study of Korean patients with migraine. Sci Rep 13:14825. doi: 10.1038/s4159802342110-4.
24. Hong JB, Lange KS, Fitzek M, Overeem LH, Triller P, Siebert A, Reuter U, Raffaelli (2023) Impact of a reimbursement policy change on treatment with erenumab in migraine - a real-world experience from Germany. J Headache Pain 24:144. doi: 10.1186/s10194-023-01682-2.
25. Lee HC, Cho S, Kim BK (2023) Predictors of response to galcanezumab in patients with chronic migraine: a real-world prospective observational study. Neurol Sci 44:2455-2463. doi: 10.1007/s10072023066832.
26. Hong, J.B.; Lange, K.S.; Overeem, L.H.; Triller, P.; Raffaelli, B.; Reuter, U (2023) A Scoping Review and Meta-Analysis of Anti-CGRP Monoclonal Antibodies: Predicting Response. Pharmaceuticals 16:934. doi.org/10.3390/ ph16070934
27. Barbanti P, Egeo G, Aurilia C, Altamura C, d'Onofrio F, Finocchi C, Albanese M, Aguggia M, Rao R, Zucco M, Frediani F, Filippi M, Messina R, Cevoli S, Carnevale A, Fiorentini G, Messina S, Bono F, Torelli P, Proietti S, Bonassi S, Vernieri F; Italian Migraine Registry study group (2022) Predictors of response to anti-CGRP monoclonal antibodies: a 24-week, multicenter, prospective study on 864 migraine patients. J Headache Pain 23:138. doi: 10.1186/s10194022014986.
28. Muñoz-Vendrell A, Campoy S, Caronna E, Alpuente A, Torres-Ferrus M, Nieves Castellanos C, Olivier M, Campdelacreu J, Prat J, Camiña Muñiz J, Molina Martínez FJ, Mínguez-Olaondo A, Ruibal Salgado M, Santos Lasaosa S, Navarro Pérez MP, Morollón N, López Bravo A, Cano Sánchez LM, García-Sánchez SM, García-Ull J, Rubio-Flores L, Gonzalez-Martinez A, Quintas S, Echavarría Íñiguez A, Gil Luque S, Castro-Sánchez MV, Adell Ortega V, García Alhama J, Berrocal-Izquierdo N, Belvís R, Díaz-Insa S, Pozo-Rosich P, Huerta-Villanueva M (2023) Effectiveness and safety of anti-CGRP monoclonal antibodies in patients over 65 years: a real-life multicentre analysis of 162 patients. J Headache Pain 24:63. doi: 10.1186/s10194023015852.
29. Mechtler L, Saikali N, McVige J, Hughes O, Traut A, Adams AM (2022) Real-World Evidence for the Safety and Efficacy of CGRP Monoclonal Antibody Therapy Added to OnabotulinumtoxinA Treatment for Migraine Prevention in Adult Patients With Chronic Migraine. Front Neurol 12:788159. doi: 10.3389/fneur.2021.788159.
30. Blumenfeld AM, Frishberg BM, Schim JD, Iannone A, Schneider G, Yedigarova L, Manack Adams A (2021) Real-World Evidence for Control of Chronic Migraine Patients Receiving CGRP Monoclonal Antibody Therapy Added to OnabotulinumtoxinA: A Retrospective Chart Review Pain Ther 10:809-826. doi: 10.1007/s4012202100264x.
31. Messina R, Huessler EM, Puledda F, Haghdoost F, Lebedeva ER, Diener HC (2023) safety and tolerability of monoclonal antibodies targeting the CGRP pathway and gepants in migraine prevention: A systematic review and network meta-analysis. Cephalalgia 43:3331024231152169. doi: 10.1177/s03331024231152169.

**Declarations**

**Ethics approval and consent to participate**:

The study protocol was approved by the ethics committee of Hospital Clinic, Barcelona, Spain. Register number HCB/2021/1327 and written informed consent was obtained from all participants.

**Consent for publication**

Not applicable

**Availability of data and materials**

Upon request, the corresponding author will provide the necessary data and materials on SSPS supported archive to interested researchers for the purpose of academic scrutiny, reproducibility, and further scientific investigation.

**Competing interests**

Within the past 24 months,

VO has received Speaker/travel grants/ clinical trials from Teva, Abbvie, Eli Lilly, Lundbeck, Pfizer and Biohaven.

ALG has received research funding from the Regional Health Administration (Gerencia Regional de Salud SACYL) in Castilla y Leon, Spain. Speaker/travel grants/ clinical trials from Teva, Abbvie, Amgen, Eli Lilly, Lundbeck, Novartis, Pfizer and Biohaven.

ALG has received research funding from the Regional Health Administration (Gerencia Regional de Salud SACYL) in Castilla y Leon, Spain. Speaker/travel grants/ clinical trials from Teva, Abbvie, Amgen, Eli Lilly, Lundbeck, Novartis, Pfizer and Biohaven.

The remaining authors report no competing interests.

**Funding**

None

### Authors and Affiliations

1- Headache Unit. Neurology Department. Hospital Clinic. Univesitat de Barcelona. IDIBAPS. Barcelona. Spain.

2- Neurology Department. Hospital Universitario Cruces. Bilbao. Spain.

3- Neurology Department. Hospital Universitario Central de Asturias. Oviedo, Spain.

4- Neurology Department. Hospital Universitario de Navarra. Spain.

5- Neurology Department. Hospital Parc Tauli. Sabadell. Barcelona. Spain

6- Neurology Department. Hospital Universitario de Galdakao-Usansolo. Bilbao. Spain.

7- Neurology Department. Hospital Universitario de Basurto. Bilbao. Spain

8- Neurology Department. Hospital Universitario de Donostia. San Sebantian. Spain

9- Neurology Department. Hospital del Mar. Department of Medicine and Life

Sciences, Universitat Pompeu Fabra (UPF), Barcelona, Spain.

10- Neurology Department. Araba University Hospital. Bioaraba. Osakidetza Basque Health Service. Spain.

11- Headache Unit, Neurology Department. Hospital Reina Sofía, Tudela de Navarra. Aragon Institute for Health Research (IIS Aragón), Zaragoza. Spain.

12- Faculty of Health and Life Sciences. Universitat Pompeu Fabra. Barcelona. Spain.

13- Headache Unit. Neurology Department. Hospital Clinico Universitario. Department of Medicine, University of Valladolid. Spain

**Authors' contributions**

All authors had full access to and take responsibility for the integrity and accuracy of the data analysis. VO and DGA conceptualized and designed the study. All authors were responsible for data acquisition, analysis, and interpretation. VO contributed to data quality control. DGA, VO and JCGM drafted the manuscript, while all authors provided critical revisions for important intellectual content.

**Acknowledgements**

Not applicable

**Abbreviations**

BCF: Baseline carried-forward

BMI: Body mass index

CM: Chronic migraine

CGRP-mAb: Calcitonin gene-related peptide monoclonal antibody

FDR: False discovery rate

HDM: Headache days per month

HFEM: High frequency episodic migraine

ITT: Intention-to-treat.

LOCF: Last-observation carried-forward

MDM: Migraine days per month

OnabotA : onabotulinumtoxin A

PP: Per protocol

RCTs: Randomized clinical trials

RWE: Real wold evidence

TEAE: Treatment emergent adverse events

Supplementary table 1: Comparison in headache days per month during all the timepoints between patients with and without each subgroup.

|  | Age >65 years | Concomitant onabotA | Daily headache | Chronic pain | Fibromyalgia | Treatment resistance |
| --- | --- | --- | --- | --- | --- | --- |
| Baseline | **0.045** | 0.365 | **<0.001** | **<0.001** | **<0.001** | 0.744 |
| Weeks 8-12 PP | 0.435 | **0.002** | **<0.001** | **<0.001** | **<0.001** | 0.502 |
| Weeks 8-12 BCF | 0.539 | **0.004** | **<0.001** | **<0.001** | **<0.001** | 0.497 |
| Weeks 20-24 PP | 0.172 | **0.023** | **<0.001** | **0.016** | **0.001** | 0.554 |
| Weeks 20-24 BCF | 0.463 | **0.033** | **<0.001** | **<0.001** | **<0.001** | 0.667 |
| Weeks 20-24 LOCF | 0.600 | **0.011** | **<0.001** | **<0.001** | **<0.001** | 0.689 |
| Weeks 44-48 PP | 0.143 | 0.254 | **<0.001** | **0.001** | **<0.001** | 0.589 |
| Weeks 44-48 BCF | 0.679 | 0.090 | **<0.001** | **<0.001** | **<0.001** | 0.566 |
| Weeks 44-48 LOCF | 0.918 | 0.029 | **<0.001** | **<0.001** | **<0.001** | 0.617 |

Supplementary table 2: Logistic regression analysis of the variables that were associated with a 50% responder rate between weeks 20-24. Values in bold denote statistical significance.

| **Variable** | **Type of**  **analysis** | **Odds ratio** | FDR-adjusted *P* value |
| --- | --- | --- | --- |
| Age | Univariate | 1.013 (95%CI: 1.003-1.024) | **0.010** |
|  | Multivariate | 1.016 (95%CI: 1.005-1.026) | **0.009** |
| Female sex | Univariate | 1.004 (05% CI: 0.728-1.383) | 0.983 |
| Chronic migraine | Univariate | 1.643 (95%CI: 1.232-2.190) | **0.001** |
|  | Multivariate | 1.326 (95%CI: 0.992-1.871) | 0.112 |
| Psychiatric  comorbidity | Univariate | 1.193 (95%CI: 1.014-1.404) | **0.034** |
|  | Multivariate | 1.215 (95%CI: 0.932-1.584) | 0.226 |
| Other painful syndromes | Univariate | 1.113 (95%CI: 0.950-1.305) | 0.185 |
|  | Multivariate | 0.933 (95%CI: 0.710-1.226) | 0.742 |
| Fibromyalgia | Univariate | 1.083 (95%CI: 0.924-1.271) | 0.324 |
| Duration of migraine disease | Univariate | 1.006 (95%CI: 0.991-1.022 | 0.412 |
| Prior number of preventives | Univariate | 0.998 (95%CI: 0.926-1.077) | 0.967 |
| Concomitant use of Botulinum toxin | Univariate | 1.121 (95%CI: 0.977-1.286) | 0.102 |
|  | Multivariate | 1.011 (95%CI: 0.822-1.245) | 0.915 |
| Daily headache at baseline | Univariate | O.585 (95%CI: 0.451-0.760) | <0.001 |
|  | Multivariate | 0.619 (95%CI: 0.469-0.817) | **0.006** |

CI: Confidence interval. Values in bold denote statistical signification. FDR: False discovery rate.

Supplementary table 3: Linear regression analysis about the variables associated with a different change in headache days per month between weeks 20-24, compared with the baseline. Analysis done per intention-to-treat.

| **Variable** | **Type of**  **analysis** | **B coefficient and 95% confidence interval** | FDR-adjusted *P* value |
| --- | --- | --- | --- |
| Age | Univariate | 0.067 (95% CI: 0.025 – 0.109) | 0.002 |
|  | Multivariate | 0.068 (95% CI: 0.018 – 0.119) | **0.014** |
| Female sex | Univariate | -0.387 (95% CI: -1.741 – 0.967) | 0.575 |
| Chronic migraine | Univariate | 2.738 (95% CI: 1.550 – 3.926) | <0.001 |
|  | Multivariate | -1.253 (95% CI: -2.778 – 0.272) | 0.125 |
| Psychiatric  comorbidity | Univariate | -1.132 (95% CI: -2.215 – -0.050) | 0.040 |
|  | Multivariate | -1.587 (95% CI: -2.636 - -0.538) | **0.009** |
| Other painful syndromes | Univariate | -0.110 (95% CI: -1.395 – 1.175) | 0.866 |
| Fibromyalgia | Univariate | -0.320 (95% CI: 2.051 – 1.411) | 0.717 |
| Duration of migraine disease | Univariate | 0.016 (95% CI: -0.050 – 0.083) | 0.633 |
| Prior number of  preventives | Univariate | 0.077 (95% CI: -0.241 – 0.395) | 0.634 |
| Treatment resistance | Univariate | 0.702 (95% CI: -1.052 – 2.456) | 0.432 |
| Age > 65 years | Univariate | 1.633 (95% CI: 0.037 – 3.228) | 0.045 |
|  | Multivariate | -0.261 (95% CI: -2.178 – 1.657) | 0.790 |
| Concomitant use of  Botulinum toxin | Univariate | -1.229 (95% CI: -2.566 – 0.109) | 0.072 |
|  | Multivariate | -1.195 (95% CI: -2.481 – 0.091) | 0.069 |
| Headache days per month at baseline | Univariate | 0.271 (95% CI: 0.208 – 0.334) | <0.001 |
|  | Multivariate | 0.451 (95% CI: 0.319 – 0.583) | **<0.001** |
| Daily headache  at baseline | Univariate | 2.902 (95% CI: 1.832 – 3.972) | <0.001 |
|  | Multivariate | -2.718 (95% CI: -4.568 - -0.869) | **0.009** |

Supplementary table 4: Linear regression analysis about the variables associated with a change in headache days per month between weeks 44-48 compared with the baseline. Analysis done per intention-to-treat.

| **Variable** | **Type of**  **analysis** | **B coefficient and 95% confidence interval** | FDR-adjusted *P* value |
| --- | --- | --- | --- |
| Age | Univariate | 0.042 (95% CI: -0.002 – 0.096) | 0.061 |
|  | Multivariate | 0.042 (95% CI: -0.011 – 0.095) | 0.168 |
| Female sex | Univariate | -0.177 (95% CI: -1.595 – 1.241) | 0.806 |
| Chronic migraine | Univariate | 2.599 (95% CI: 1.353 – 3.846) | <0.001 |
|  | Multivariate | -1.779 (95% CI: -3.376 - -0.181) | 0.0507 |
| Psychiatric  comorbidity | Univariate | -1.375 (95% CI: -2.508 - -0.242) | 0.017 |
|  | Multivariate | -1.932 (95% CI: -3.030 - -0.833) | **0.003** |
| Other painful  syndromes | Univariate | -0.217 (95% CI: -1.563 – 1.128) | 0.751 |
| Fibromyalgia | Univariate | -0.939 (95% CI: -2.752 – 0.873) | 0.309 |
| Duration of  migraine disease | Univariate | -0.016 (95% CI: -0.086 – 0.054) | 0.656 |
| Prior number of  preventives | Univariate | 0.087 (95% CI: -0.246 – 0.420) | 0.608 |
| Treatment resistance | Univariate | 0.868 (95% CI: -0.969 – 2.705) | 0.354 |
| Age > 65 years | Univariate | 1.128 (95% CI: -0.545 – 2.801) | 0.186 |
|  | Multivariate | -0.284 (95% CI: -2.293 – 1.724) | 0.781 |
| Concomitant use of  Botulinum toxin | Univariate | -0.667 (95% CI: -2.070 – 0.735) | 0.351 |
|  | Multivariate | -0.615 (95% CI: -1.962 – 0.732) | 0.438 |
| Headache days per month at baseline | Univariate | 0.299 (95% CI: 0.233 – 0.364) | <0.001 |
|  | Multivariate | 0.473 (95% CI: 0.335 – 0.611) | **<0.001** |
| Daily headache  at baseline | Univariate | 3.523 (95% CI: 2.408 – 4.638) | <0.001 |
|  | Multivariate | -0.284 (95% CI: -4.114 - -0.241) | 0.0507 |
